# Differences in tobacco smoking prevalence and frequency between adolescent Palestine refugee and non-refugee populations in Jordan, Lebanon, Syria, and the West Bank: cross-sectional analysis of the Global Youth Tobacco Survey

**DOI:** 10.1186/s13031-016-0087-4

**Published:** 2016-10-05

**Authors:** Mohammed Jawad, Ali Khader, Christopher Millett

**Affiliations:** 1 Department of Primary Care and Public Health, School of Public Health, Imperial College London, London, W6 8RP UK; 2 United Nations Relief and Works Agency (UNRWA) for Palestine refugees in the Near East, Amman, Jordan

**Keywords:** Tobacco, Cigarettes, Waterpipe, Refugee, Adolescents

## Abstract

**Background:**

Evidence is conflicting as to the whether tobacco smoking prevalence is higher in refugee than non-refugee populations. The aim of this study was to compare the prevalence and frequency of tobacco smoking in Palestine refugee and non-refugee adolescent populations in the Middle East.

**Methods:**

We conducted a cross-sectional analysis of the Global Youth Tobacco Survey (GYTS) conducted in Jordan, Lebanon, Syria, and the West Bank among adolescent Palestine refugees and non-refugees. Age- and sex-adjusted regression models assessed the association between refugee status and current (past-30 day) tobacco use prevalence and frequency.

**Results:**

Prevalence estimates for current tobacco smoking were similar between Palestine refugee and non-refugee groups in Jordan (26.7 % vs. 24.0 %), Lebanon (39.4 % vs. 38.5 %), and the West Bank (39.5 % vs. 38.4 %). In Syria, Palestine refugees had nearly twice the odds of current tobacco smoking compared to non-refugees (23.2 % vs. 36.6 %, AOR 1.96, 95 % CI 1.46–2.62). Palestine refugees consumed more cigarettes per month than non-refugees in Lebanon (β 0.57, 95 % CI 0.17–0.97) and Palestine refugees consumed more waterpipe tobacco per month than non-refugees in Syria (β 0.40, 95 % CI 0.19–0.61) and the West Bank (β 0.42, 95 % CI 0.21–0.64).

**Conclusions:**

Current tobacco smoking prevalence is in excess of 20 % in both adolescent Palestine refugee and non-refugee populations in Middle Eastern countries, however Palestine refugees may smoke tobacco more frequently than non-refugees. Comparison of simple prevalence estimates may therefore mask important differences in tobacco use patterns within population groups.

## What is already known on this subject?

- Important sociodemographic disparities exist in tobacco smoking prevalence

## What important gaps in knowledge exist on this topic?

- The relationship between tobacco use and refugee status is understudied and unclear

## What this study adds?

- Refugees have a higher tobacco smoking prevalence in Syria, and smoke tobacco more frequently in Lebanon, Syria and the West Bank
- Assessment of disparities in tobacco smoking should extend beyond simple prevalence measures and include measures of smoking frequency

## Background

An estimated six million people die each year from conditions attributable to tobacco use; 80 % of which occur in low and middle income countries [1]. This figure is estimated to rise to 8 million by the year 2030, resulting in calls from the United Nations General Assembly for a global movement to achieve a 30 % relative reduction in current tobacco use by 2025 [2]. The Middle East is one of two regions worldwide that will continue to see an increase in tobacco use should current tobacco control policies stay as they are [3]. The Middle East is also experiencing a surge in waterpipe smoking: a predominantly flavoured form of tobacco consumption where charcoal-heated tobacco smoke is drawn through an apparatus containing water [4]. The most recent estimates from the Global Youth Tobacco Survey (GYTS) suggests that past-30 day waterpipe tobacco use among adolescents is highest in Lebanon (36.9 %), followed by the West Bank (32.7 %), Syria (20.1 %) and Jordan (18.9 %) [5].

Important sociodemographic disparities exist in tobacco use, including disparities by ethnicity or race [6]. One understudied area related to this is the relationship between tobacco use and refugee status. Refugees are considered among the most vulnerable of population groups. The 1951 Refugee Convention defines a refugee as someone who “owing to a well-founded fear of being persecuted for reasons of race, religion, nationality, membership of a particular social group or political opinion, is outside the country of his nationality, and is unable to, or owing to such fear, is unwilling to avail himself of the protection of that country” [7]. Over 5 million Palestine refugees live in the Middle East and are registered with the United Nations Relief and Works Agency (UNRWA) [8].

A recent systematic review on tobacco use among those affected by armed conflict suggested that evidence addressing the association between refugee status and tobacco use is limited and conflicting [9]. Furthermore, most studies addressing this topic were found to be of low methodological quality and conducted in European or US settings, resulting in a call for substantially more research. For example, a 10 year review of refugees in a western US state showed that Iranian and Vietnamese refugees had increased odds of tobacco use while Ukrainian refugees had lower odds of tobacco use compared with the general population [10]. Lower prevalence of tobacco use among refugee than non-refugee populations has also been documented among adolescent refugees living in an urban Canadian city [11] and among Cambodian women living in an eastern US state [12]. A study conducted in the Lebanese capital Beirut showed that elderly Palestine refugees reported a higher prevalence of current cigarette use (34.3 % vs. 28.2) but a similar number of cigarette pack years and a later age of cigarette initiation than elderly non-refugees in neighbouring areas [13].

There is a need to better assess the relationship between refugee status and tobacco use to add to the existing body of evidence. The Middle East is an ideal place to study this relationship given nearly 50 % of the world’s refugees are Middle Eastern [8] and it is a region of increasing tobacco use [1]. The Palestine refugee population is the largest sub-group of refugees in this region, reaching over 5 million in number, and have been forcibly displaced since 1948 [14]. A third of Palestine refugees live in one of 59 camps in Jordan, Lebanon, Syria, Gaza and the West Bank, while two thirds live in cities, towns or villages in these countries, many of which are close to refugee camps [14]. Previous assessments of this and other conflict-affected populations have been limited to simple tobacco prevalence estimates, with no indication of how frequency of use may differ between refugee and non-refugee populations [15, 16]. The aim of this study was therefore to compare the prevalence and frequency of tobacco smoking in Palestine refugee and non-refugee populations in the Middle East.

## Methods

### Sample and data

We conducted a secondary analysis of the GYTS [17]. The GYTS is a cross-sectional, self-administered survey considered the gold standard for tobacco surveillance among school students typically aged 13–15 years. Standardised methodologies are implemented across countries to produce regionally- or nationally-representative data. GYTS participants are selected in a two-stage cluster design, where schools are selected with a probability proportional to their sizes and classes are selected with equal probability. Individual level analyses weights, which include sample selection and post-stratification factor, were provided for each country. More details on the GYTS methodology can be found elsewhere [18]. Given the GYTS is routinely available this study was exempt from ethics approval.

Datasets used in this study were the GYTS for Palestine refugee school children aged 13–15 years enrolled in UNRWA schools in Jordan, Lebanon, Syria and the West Bank (all conducted in 2008) and the GYTS for non-refugees in Jordan (conducted in 2009), Lebanon (2011), Syria (2010) and the West Bank (2009). The GYTS sampling methodologies for Palestine refugee (UNRWA) schools and non-refugee schools were identical in each country.

### Measures

The primary outcome measure was current tobacco smoking prevalence, defined as past-30 day cigarette or waterpipe use. We also stratified this by cigarette-only, waterpipe-only, and dual use. Secondary outcome measures included the frequency of cigarettes or waterpipes smoked per month, calculated by multiplying the values of the questions “During the past 30 days (1 month), on how many days did you smoke [cigarettes/waterpipe]?” and “During the past 30 days (1 month), on the days you smoked, how many [cigarettes/waterpipes] did you usually smoke?”. Independent variables included population type (Palestine refugee or non-refugee), age, and sex. No other socioeconomic variables were available in these publically available datasets [17].

### Statistical analysis

We calculated nationally-representative estimates for current tobacco smoking prevalence (including cigarette-only, waterpipe-only, and dual use) for Palestine refugee and non-refugee populations in Jordan, Lebanon, Syria, and the West Bank, reporting 95 % confidence intervals (CIs). For each country we constructed regression models which assessed the association between refugee status with current tobacco smoking (logistic regression) and frequency of use (linear regression). The variables for frequency of cigarette and waterpipe use were logarithmically transformed prior to its inclusion in linear regression models to account for its skewed distribution. All models were adjusted for the age and sex of respondents. Survey weights were used to account for the multi-stage design of the GYTS. Analyses were conducted on Stata 12.0 (StataCorp).

## Results

### Sample characteristics

Table 1 presents sample characteristics of Palestine refugee and non-refugee populations in Jordan, Lebanon, Syria, and the West Bank. In line with the target age group for the GYTS, between 74 and 81 % of each population group in each country were aged 13–15 years. The exception to this was the West Bank GYTS, which appeared to sample a slightly younger non-refugee population (only 68 % aged 13–15 years). The proportion males varied between 40 % (Jordan non-refugee and West Bank refugee samples) and 51 % (Jordan refugee sample).

**Table 1:** Sample characteristics of Palestine refugee and non-refugee adolescents in the Middle East

	Jordan		Lebanon		Syria		West Bank	
	Refugee	Non-refugee	Refugee	Non-refugee	Refugee	Non-refugee	Refugee	Non-refugee
Age in years, % (*n*)								
<12	*5.0 (69)*	*6.4 (114)*	*2.1 (34)*	*2.1 (51)*	*3.0 (48)*	*4.4 (72)*	*7.3 (107)*	*3.6 (75)*
12	*8.6 (123)*	*18.3 (322)*	*6.6 (107)*	*11.0 (264)*	*9.8 (153)*	*12.1 (228)*	*11.5 (180)*	*16.5 (368)*
13	*26.2 (370)*	*31.8 (568)*	*24.5 (384)*	*24.8 (571)*	*30.5 (479)*	*29.0 (547)*	*26.1 (389)*	*22.4 (443)*
14	*31.1 (477)*	*27.9 (570)*	*29.6 (425)*	*28.7 (592)*	*30.8 (495)*	*32.5 (391)*	*33.1 (447)*	*23.9 (439)*
15	*23.1 (375)*	*13.9 (283)*	*22.3 (368)*	*21.4 (408)*	*20.0 (330)*	*13.0 (211)*	*19.0 (277)*	*21.9 (391)*
16	*5.3 (82)*	*1.5 (28)*	*11.9 (204)*	*8.4 (189)*	*5.3 (87)*	*6.4 (172)*	*2.3 (33)*	*10.4 (192)*
>16	*0.7 (11)*	*0.3 (6)*	*3.1 (54)*	*3.5 (81)*	*0.7 (11)*	*2.4 (10.4)*	*0.7 (11)*	*1.3 (24)*
Sex, % (*n*)								
Female	*49.4 (54.6)*	*59.7 (1094)*	*54.1 (48.5)*	*52.6 (1129)*	*49.8 (811)*	*51.4 (973)*	*59.7 (860)*	*52.9 (1022)*
Male	*50.6 (45.4)*	*40.3 (750)*	*45.9 (51.5)*	*47.4 (1074)*	*50.2 (769)*	*48.7 (640)*	*40.4 (515)*	*47.1 (931)*
Year of survey								
	*2008*	*2009*	*2008*	*2011*	*2008*	*2010*	*2008*	*2009*

### Prevalence of current tobacco use

Table 2 presents the prevalence and frequency of current tobacco smoking in Palestine refugee and non-refugee groups. The prevalence of current tobacco smoking was similar between Palestine refugee and non-refugee groups in Jordan (26.7 % vs. 24.0 %), Lebanon (39.4 % vs. 38.5 %), and the West Bank (39.5 % vs. 38.4 %). A similar pattern was seen for current cigarette-only smoking, current waterpipe-only smoking, and current dual smoking for these three countries. In Syria, however, Palestine refugees had a higher prevalence of current tobacco smoking (36.6 % vs. 23.2 %), and current waterpipe-only smoking (22.7 % vs. 15.1 %) compared to non-refugees.

**Table 2:** Prevalence and frequency of current tobacco smoking in Palestine refugees and non-refugees

	Jordan		Lebanon		Syria		West Bank	
	Non-refugee	Refugee	Non-refugee	Refugee	Non-refugee	Refugee	Non-refugee	Refugee
Current tobacco smoking								
Prevalence, % (95 % CI)	*24.0 (19.3, 29.6)*	*26.7 (19.9, 34.9)*	*38.5 (33.4, 43.3)*	*39.4 (32.1, 47.1)*	*23.2 (18.5, 28.7)*	*36.6 (31.4, 42.3)*	*38.4 (29.5, 48.2)*	*39.5 (31.3, 48.2)*
Cigarettes per month, median (IQR)	*4.0 (0.8, 50.8)*	*4.0 (0.8, 26.3)*	*1.5 (0.8, 14.8)*	*5.3 (0.8, 55.4)*	*1.5 (0.8, 14.5)*	*4.0 (0.8, 14.5)*	*4.0 (0.8, 50.8)*	*4.0 (1.5, 50.8)*
Waterpipes per month, median (IQR)	*1.5 (0.8, 4.0)*	*1.5 (0.8, 7.3)*	*2.0 (0.8, 14.0)*	*2.0 (0.8, 14.0)*	*0.8 (0.8, 3.8)*	*1.5 (0.8, 7.5)*	*1.5 (0.8, 7.5)*	*2.0 (0.8, 14.0)*
Current cigarette-only smoking								
Prevalence, % (95 % CI)	*4.4 (3.0, 6.2)*	*5.9 (4.1, 8.5)*	*1.7 (1.1, 2.6)*	*2.9 (1.9, 4.4)*	*2.8 (1.6, 4.8)*	*5.4 (3.7, 7.8)*	*5.6 (4.1, 7.6)*	*7.8 (6.1, 10.0)*
Cigarettes per month, median (IQR)	*1.8 (0.8, 26.3)*	*2.0 (0.8, 15.0)*	*0.8 (0.8, 2.0)*	*3.9 (0.8, 24.8)*	*1.5 (0.8, 7.5)*	*2.0 (0.8, 7.5)*	*1.5 (0.8, 7.5)*	*1.5 (0.8, 14.0)*
Current waterpipe-only smoking								
Prevalence, % (95 % CI)	*12.3 (10.2, 14.8)*	*12.5 (10.9, 14.2)*	*24.9 (20.4, 30.0)*	*26.8 (21.4, 32.9)*	*15.1 (12.4, 18.3)*	*22.7 (19.9, 25.8)*	*16.9 (14.0, 20.1)*	*16.4 (14.4, 18.6)*
Waterpipes per month, median (IQR)	*0.8 (0.8, 1.5)*	*0.8 (0.8, 4.0)*	*0.8 (0.8, 4.0)*	*1.5 (0.8, 5.3)*	*0.8 (0.8, 2.0)*	*1.5 (0.8, 4.0)*	*0.8 (0.8, 2.0)*	*1.5 (0.8, 5.3)*
Current dual smoking								
Prevalence, % (95 % CI)	*7.3 (5.2, 10.1)*	*8.4 (4.9, 14.0)*	*11.9 (10.4, 13.6)*	*9.7 (6.2, 15.0)*	*5.3 (3.8, 7.4)*	*8.5 (6.2, 11.7)*	*15.9 (10.4, 23.6)*	*15.2 (10.0, 22.6)*
Cigarettes per month, median (IQR)	*4.0 (0.8, 85.8)*	*4.0 (0.8, 26.3)*	*1.5 (0.8, 30.0)*	*7.4 (1.5, 73.9)*	*2.0 (0.8, 26.3)*	*4.0 (0.8, 26.3)*	*5.3 (1.5, 60.0)*	*7.3 (1.5, 72.9)*
Waterpipes per month, median (IQR)	*3.8 (0.8, 14.0)*	*3.8 (0.8, 7.5)*	*7.3 (1.5, 50.8)*	*7.5 (1.5, 30.0)*	*1.8 (0.8, 4.0)*	*3.8 (0.8, 14.5)*	*3.8 (0.8, 14.5)*	*7.3 (1.5, 24.5)*

*Abbreviations*: *95 % CI* 95 % confidence interval, *IQR* interquartile range

### Association between refugee status and current tobacco use and frequency

Table 3 presents the age- and sex-adjusted correlates of current smoking prevalence and frequency. In Jordan there were no differences in all measures of tobacco smoking prevalence and frequency between Palestine refugee and non-refugee populations.

**Table 3:** Odds of current smoking prevalence and frequency in Palestine refugees and non-refugees

	Jordan		Lebanon		Syria		West Bank	
	Non-refugee	Refugee	Non-refugee	Refugee	Non-refugee	Refugee	Non-refugee	Refugee
Current tobacco smoking								
Prevalence, AOR (95 % CI)	*1.00*	*0.91 (0.67, 1.24)*	*1.00*	*1.00 (0.76, 1.31)*	*1.00*	*1.96 (1.46, 2.62)* ^*+*^	*1.00*	*1.04 (0.82, 1.32)*
Cigarettes per month, β (95 % CI)	*0.00*	*−0.35 (−0.77, 0.07)*	*0.00*	*0.57 (0.17, 0.97)^*	*0.00*	*−0.01 (−0.43, 0.41)*	*0.00*	*0.22 (−0.10, 0.54)*
Waterpipes per month, β (95 % CI)	*0.00*	*0.06 (−0.17, 0.29)*	*0.00*	*−0.03 (−0.22, 0.15)*	*0.00*	*0.40 (0.19, 0.61)* ^*+*^	*0.00*	*0.42 (0.21, 0.64)* ^*+*^
Current cigarette-only smoking								
Prevalence, AOR (95 % CI)	*1.00*	*1.06* *(0.70, 1.59)*	*1.00*	*1.65 (0.92, 2.96)*	*1.00*	2.16 (1.10, 4.24)*	*1.00*	1.53 (1.10, 2.14)*
Cigarettes per month, β (95 % CI)	*0.00*	*−0.29* *(−0.95, 0.38)*	*0.00*	1.07 (0.19, 1.96)*	*0.00*	*0.21 (−0.39, 0.81)*	*0.00*	*0.36 (−0.16, 0.88)*
Current waterpipe-only smoking								
Prevalence, AOR (95 % CI)	*1.00*	*0.88 (0.71, 1.10)*	*1.00*	*1.07 (0.74, 1.53)*	*1.00*	*1.62 (1.23, 2.14)^*	*1.00*	*0.93 (0.69, 1.24)*
Waterpipes per month, β (95 % CI)	*0.00*	*0.11 (0.86, 4.23)*	*0.00*	*0.09 (−0.11, 0.28)*	*0.00*	*0.35 (0.13, 0.58)^*	*0.00*	*0.37 (0.14, 0.61)^*
Current dual smoking								
Prevalence, AOR (95 % CI)	*1.00*	*0.91 (0.59, 1.40)*	*1.00*	*0.76 (0.52, 1.12)*	*1.00*	1.64 (1.11, 2.41)*	*1.00*	*0.94 (0.68, 1.30)*
Cigarettes per month, β (95 % CI)	*0.00*	*−0.37 (−0.92, 0.18)*	*0.00*	0.53 (0.08, 0.98)*	*0.00*	*−0.04 (−0.60, 0.53)*	*0.00*	*0.22 (−0.17, 0.62)*
Waterpipes per month, β (95 % CI)	*0.00*	*−0.15 (−0.56, 0.27)*	*0.00*	*−0.09 (−0.46, 0.28)*	*0.00*	0.54 (0.06, 1.01)*	*0.00*	*0.46 (0.12, 0.81)^*

*Abbreviations*: *AOR* adjusted odds ratio, *95 % CI* 95 % confidence interval, *β* beta coefficient

*p < 0.05; ^*p* < 0.01; ^+^ *p* < 0.001

In Lebanon there was no difference in current tobacco smoking prevalence between Palestine refugee and non-refugee populations, however Palestine refugees reported consuming more cigarettes per month than non-refugees (β 0.57, 95 % CI 0.17–0.97). Higher frequency of cigarettes per month among Palestine refugees was also found among those who reported cigarette-only and dual use.

In Syria, current tobacco smoking prevalence was higher in Palestine refugee than in non-refugee populations (AOR 1.96, 95 % CI 1.46–2.62). Furthermore, Palestine refugees reported consuming more waterpipes per month than non-refugees (β 0.40, 95 % CI 0.19–0.61). Higher frequency of waterpipes per month among Palestine refugees was also found among those who reporting waterpipe-only and dual use.

In the West Bank, there was no difference in current tobacco smoking prevalence between Palestine refugee and non-refugee populations, although cigarette-only use was higher among Palestine refugees than non-refugees (AOR 1.53, 95 % CI 1.10–2.14). Higher frequency of waterpipes per month among Palestine refugees was also found among those who reporting waterpipe-only and dual use.

## Discussion

Palestine refugees had a higher current smoking prevalence than non-refugees in Syria (for all measures of prevalence) and the West Bank (for cigarette-only smoking). Palestine refugees also had a higher frequency of use of tobacco products in Lebanon, Syria, and the West Bank. This study shows that simple prevalence estimates may mask important differences in tobacco use within population groups in areas affected by conflict. While higher smoking prevalence and frequency in refugee populations may be explained by pre-migration factors (e.g. exposure to traumatic events such as armed conflict) and factors during displacement (hardships associated with travel), given many Palestine refugees are long-term settlers post-migration factors (e.g. poor living conditions and limited life opportunities) [19, 20] may play a more pertinent role in explaining such findings. Such stressors may trigger or exacerbate mental health conditions such as post-traumatic stress disorder, thereby increasing the risk of tobacco consumption due to the perception that tobacco may alleviate stress [21]. The Palestine refugee population has been forcibly displaced since 1948, and many UNRWA camps continue to be overcrowded, have poor sanitation and generally poor living conditions [14].

The similar smoking prevalence between Palestine refugee and non-refugees in Jordan, Lebanon, and the West Bank may reflect a degree of acculturation. This is generally supported by our findings in that current tobacco prevalence between refugees and non-refugees in these countries varied only by up to 2.7 % (Jordan), whereas between-country differences in current tobacco prevalence among refugees varied by up to 12.8 % (Jordan vs. the West Bank). Similar smoking prevalence may also reflect a temporal effect; given the rising prevalence of tobacco use in the region and the fact that all refugee surveys were conducted between 1 and 3 years before their respective non-refugee populations, one may expect the time-adjusted refugee prevalence estimates to be higher than those reported in our study.

Our findings have both research and public health implications. The burden of tobacco use can be more accurately measured taking into account frequency of use rather than simple prevalence estimates. However it may be difficult to characterise waterpipe tobacco exposure accurately due to the complex and variable session lengths and sharing behaviours associated with it [22]. This is an important area for research, particularly for the Eastern Mediterranean Region where waterpipe tobacco smoking is the predominant form of tobacco use among adolescents [5]. There is a need to develop and evaluate cessation interventions, both in the context of refugee populations, and in the context of dual cigarette and waterpipe use. More research is also needed to determine better prevalence estimates in refugee adults, and factors driving differences including exposure to conflict.

This study has several limitations. The GYTS does not provide data on mental health status so we were unable to assess whether this may have influenced our findings. We were also unable to control for whether Palestine refugees lived inside or outside refugee camps, nor any other socioeconomic measure, as these variables not available on the publically available version of the GYTS. However, published reports suggest no difference in tobacco smoking prevalence between Palestine refugees living inside or outside refugee camps [15]. Outside the refugee setting, it appears both cigarette and waterpipe tobacco smoking are associated with increased wealth among women in Jordan [23]. Interestingly, it appears that the association between waterpipe tobacco smoking and high socioeconomic status remains even in societies where cigarette smoking is associated with low socioeconomic status [24]. Another limitation includes the lack of stability in the region that surveillance data may become quickly outdated; particularly with regards to Syria where several million have been externally displaced due to civil war [8]. We did not consider the effect of time on our comparisons which, as explained above, may underestimate our tobacco prevalence estimates for refugee populations. Refugees in a given country should not be considered homogenous; while our sample was limited to UNRWA-registered Palestine refugees, a significant number of Syrian and Iraqi refugees also reside in these countries and we are unable to assess patterns of tobacco use amongst them.

## Conclusions

Adolescent Palestine refugee and non-refugees have a similar prevalence of current tobacco smoking in Jordan, Lebanon, and the West Bank, however Palestine refugees smoke tobacco more frequently than non-refugees. Comparison of simple prevalence estimates may therefore mask important differences in tobacco use patterns within population groups. Ongoing tobacco surveillance is warranted and feasible, context-specific interventions for refugee populations should be developed.
